# Supplementary material for: A dirigent family protein confers variation of Casparian strip thickness and salt tolerance in maize
Source: Nat Commun. 2022 Apr 25;13:2222. doi: 10.1038/s41467-022-29809-0 (PMC9038930; doi:10.1038/s41467-022-29809-0)
Supplement: Supplementary file 1 — Supplementary Information [file 41467_2022_29809_MOESM1_ESM.pdf]

A dirigent family protein confers variation of Casparian strip thickness and salt tolerance in maize

Wang et al.

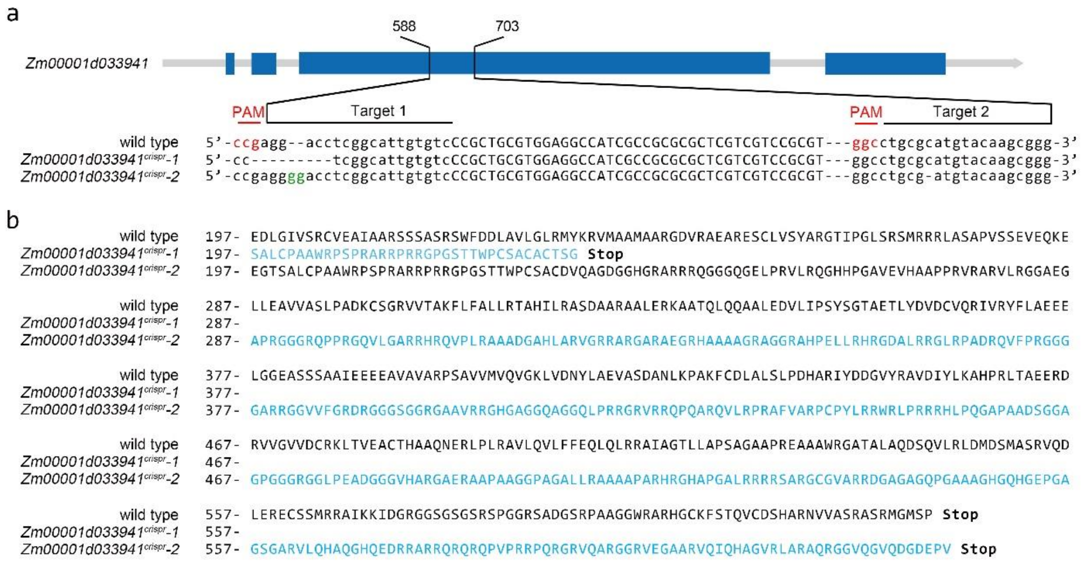

Supplementary Fig. 1. Targeted mutagenesis of *Zm00001d033941* using CRISPR-Cas9-based approach. (a) Mutations in two independent lines (*Zm00001d033941^{crispr}-1* and *Zm00001d033941^{crispr}-2*) were shown. PAM sequences were labeled in red. **(b)** Alignment of the amino acid sequences of Zm00001d033941, Zm00001d033941^{crispr}-1 and Zm00001d033941^{crispr}-2. Only the sequences flanking the mutations have been shown. The frameshifted sequences in *Zm00001d033941^{crispr}-1* and *Zm00001d033941^{crispr}-2* were highlighted in blue color. Source data are provided as a Source Data file.

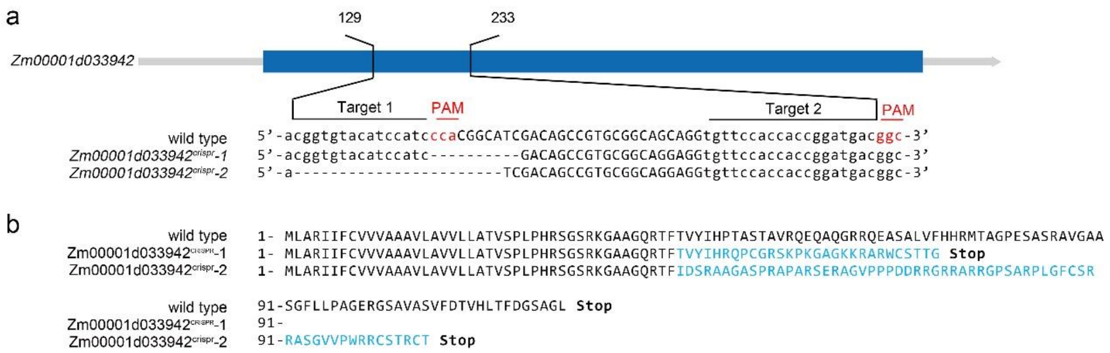

Supplementary Fig. 2. Targeted mutagenesis of *Zm00001d033942* using CRISPR-Cas9-based approach. (a) Mutations in two independent lines (*Zm00001d033942*^{crispr}*-1* and *Zm00001d033942*^{crispr}*-2*) were shown. PAM sequences were labeled in red. **(b)** Alignment of the amino acid sequences of Zm00001d033942, Zm00001d033942^{crispr}-1 and Zm00001d033942^{crispr}-2. Only the sequences flanking the mutations have been shown. The frameshifted sequences in *Zm00001d033942*^{crispr}*-1* and *Zm00001d033942*^{crispr}*-2* were highlighted in blue color. Source data are provided as a Source Data file.

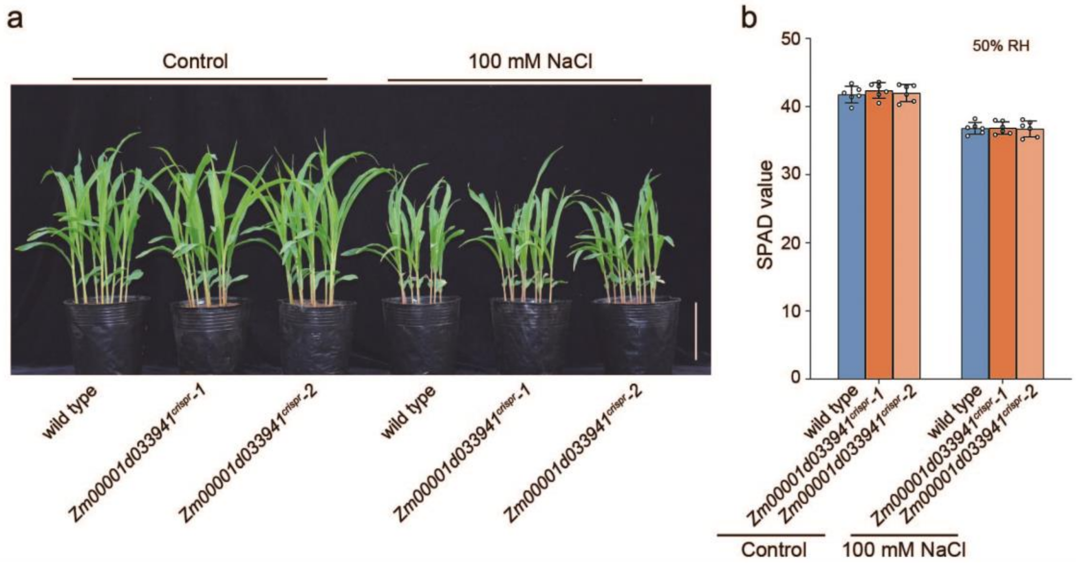

Supplementary Fig. 3. Lacking Zm00001d033941 function has undetectable effect on salt tolerance. The appearances **(a)** and SPAD values (**b**) of *Zm00001d033941^{crispr}-1*, *Zm00001d033941^{crispr}-2* and wild-type plants grown under control and salt (100 mM NaCl) condition with 50% relative humidity (RH). The results in **b** were means ± s.d. of three independent experiments. Bar in **a** equaled to 10 cm. Source data are provided as a Source Data file.

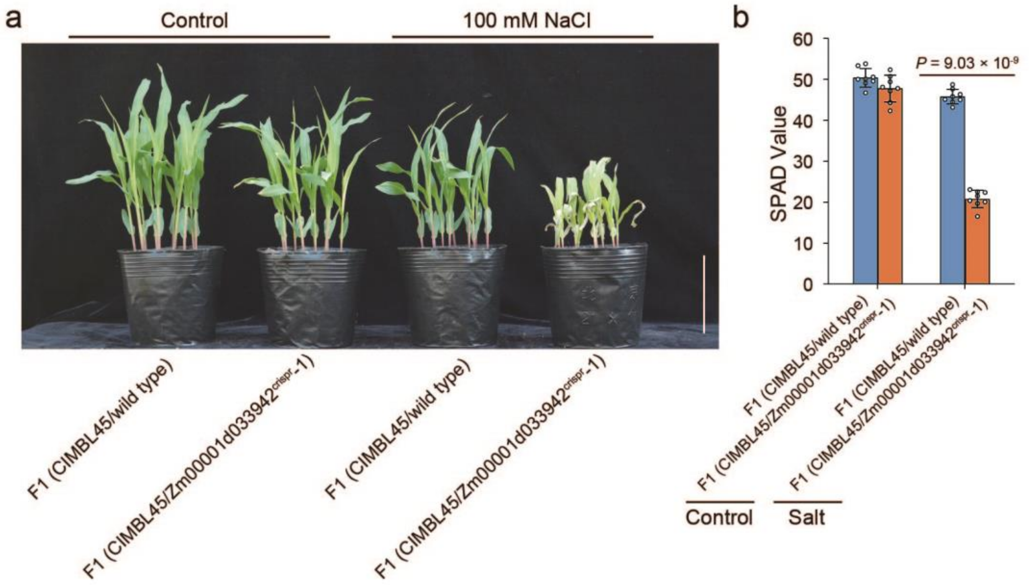

Supplementary Fig. 4. The appearances (a) and SPAD values (b) of the indicated plants grown under control and salt (100 mM NaCl) conditions with 50% relative humidity (RH). The results in **b** were means ± s.d. of three independent experiments. Statistical significances were determined by a two-sided *t*-test. Bar in **a** equaled to 10 cm. Source data are provided as a Source Data file.

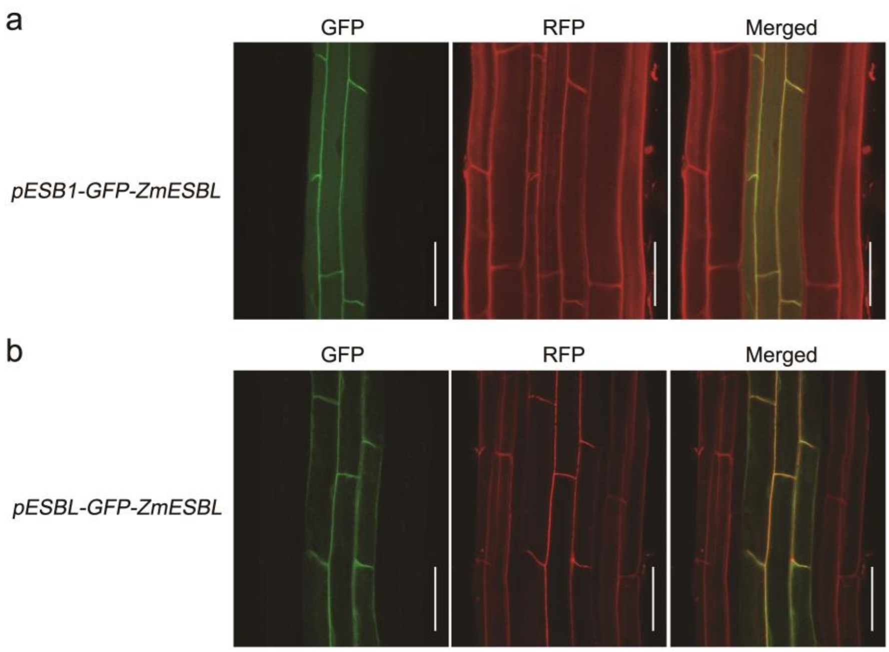

Supplementary Fig. 5. The subcellular localization of GFP-ZmESBL in Aarbidopsis endodermal cells. The subcellular location of GFP-ZmESBL protein in the Arabidopsis plants transformed with *pESB1-GFP-ZmESBL* **(a)** and *pESBL-GFP-ZmESBL* **(b)**. The red signal indicated the basic fuchsin staining of lignin, and the yellow color indicated the co-localization of GFP-ZmESBL and lignin staining in the endodermal Casparin strips. The images in **a** and **b** were representative of three independent repeats with similar results. Bars = 30 µm. Source data are provided as a Source Data file.

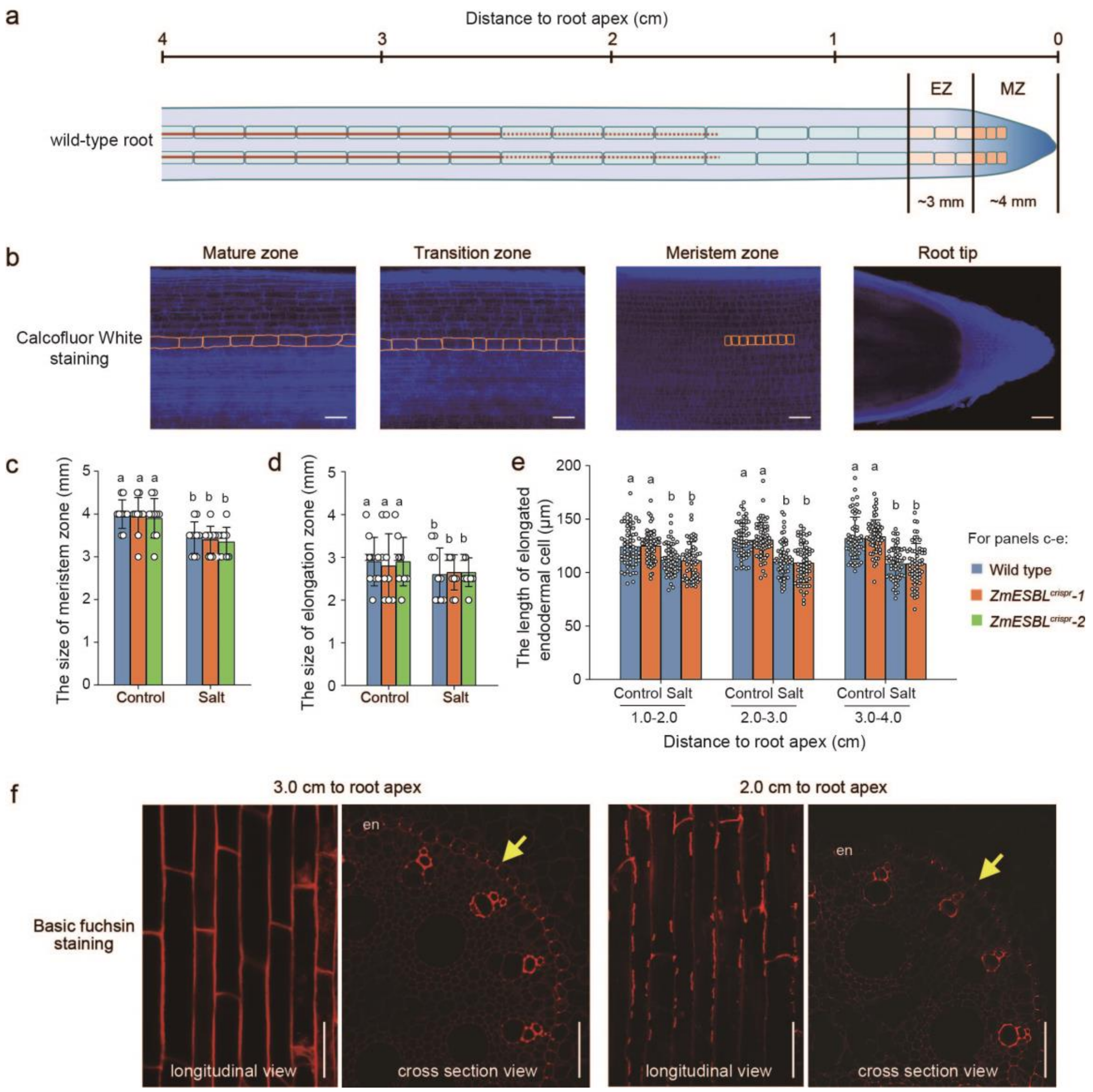

Supplementary Fig. 6. The profiles of maize primary root under salt and control conditions. (a) A graphical demonstration of the profiles of the maize primary root grown under control condition. The red dash lines indicated Casparian strip, and the solid red lines indicated the fully functional CS barrier. **(b)** A demonstration of the cell size at indicated developmental stages. Calcofluor White stains cellulose. **(c-e)** The size of meristem **(c)** and elongation **(d)** zone, and the length of the fully elongated endodermal cell **(e)**. The genotypes and treatments were as indicated, and the result indicated that the salt (100 mM NaCl) reduced the size of the meristem by ~500 µm, reduced the length of the elongation zone by ~500 µm, and reduced the length of the elongated endodermal cells by 12%. **(f)** Longitudinal and cross section view of the Casparin strips at the early (2.0 cm to root apex; ~ the 104th elongated endodermal cell) and later (3.0 cm to root apex; ~ the 184th elongated endodermal cell) root development stages. The Casparin strips were stained with basic fuchsin, and the

results indicated that maize Casparin strip starts with a discontinuous lignin deposition, and then develops into ring-like mature Casparian strips. The arrows indicated the dot-like staining of lignin at the endodermal CS domain. en, endodermis. The images in **f** were representative of three independent repeats with similar results. Data in **c-e** were means ± s.d of 9 roots (**c** and **d**) or 60 cells (**e**) from three independent batches of seedlings. Bars in **b** and **f** respectively equaled to 100 and 50 μm. Source data are provided as a Source Data file.

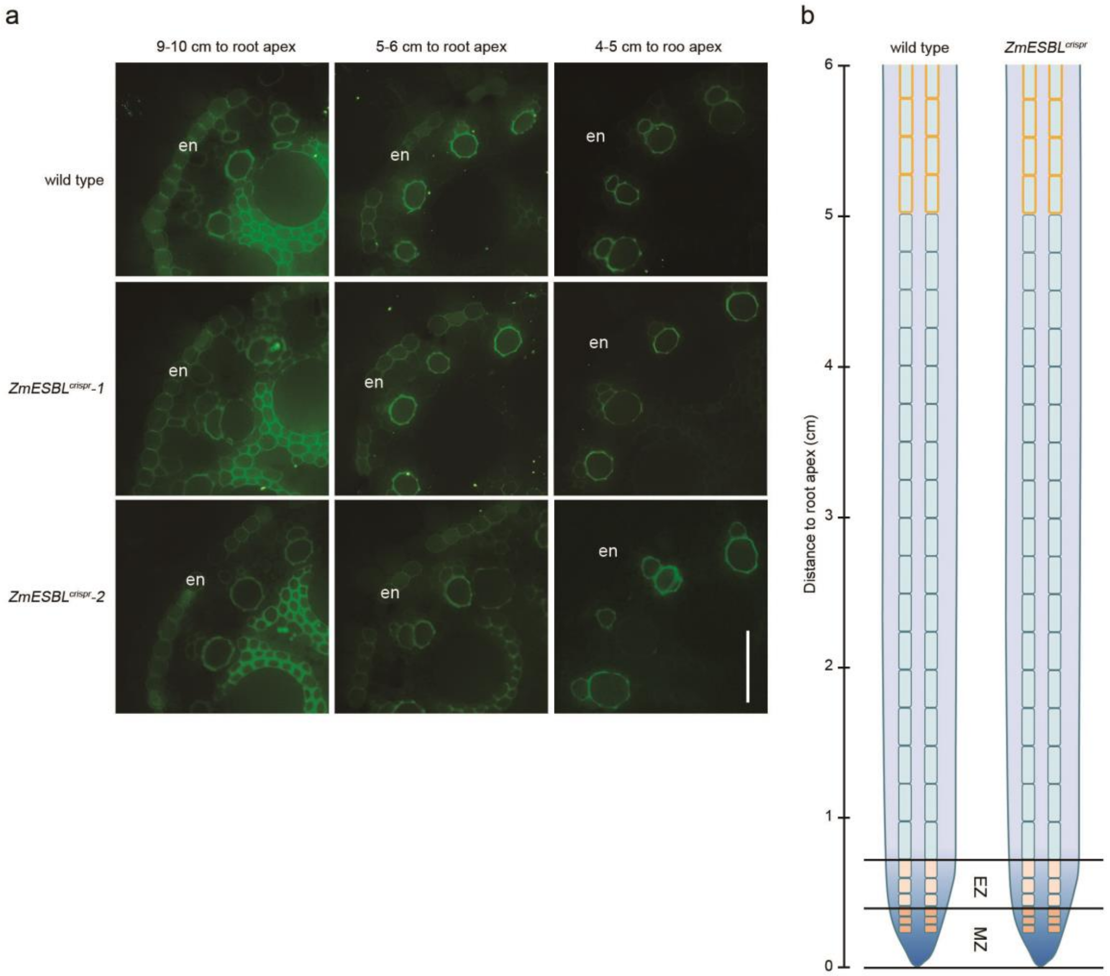

Supplementary Fig. 7. Comparison of the suberin deposition in the wild-type and *ZmESBL*^{crispr} roots. (a) The fluorol yellow 088 staining of the primary roots at the indicated locations. The images were representative of three independent repeats with similar results. **(b)** A graphical demonstration of locations where the endodermal suberin deposition starts. en, endodermis; EZ, elongation zone; MZ, meristem zone; Bar, 40 µm. Source data are provided as a Source Data file.

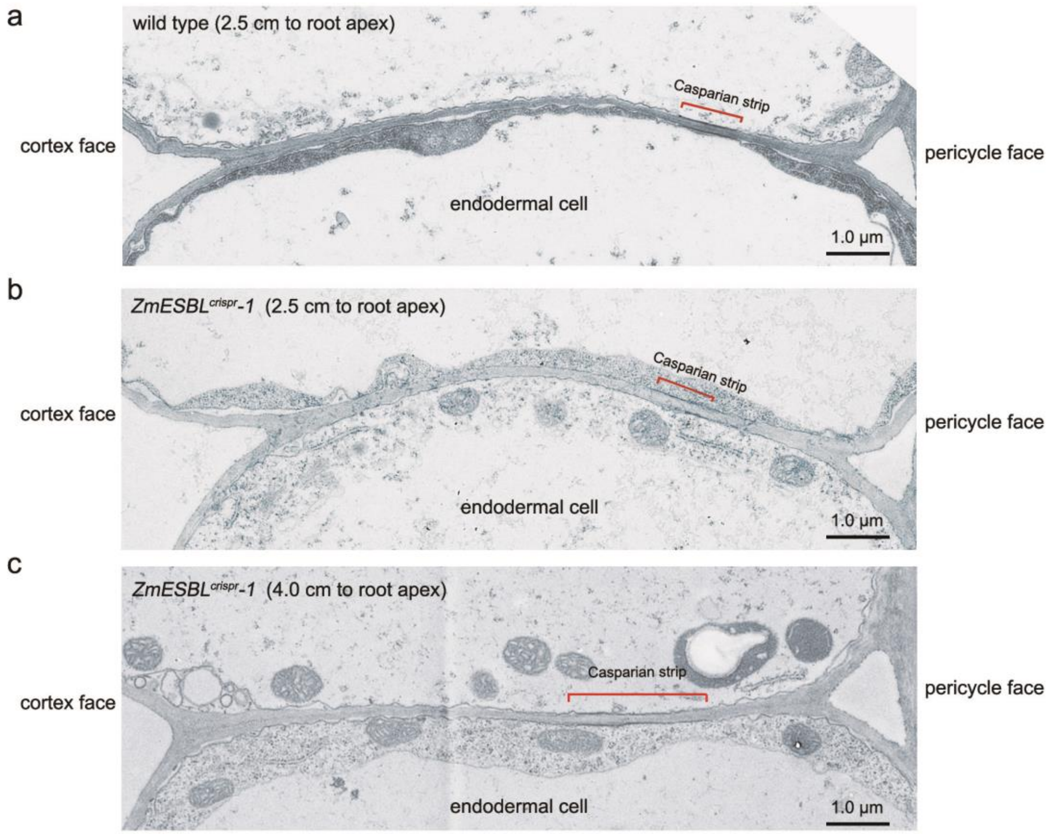

Supplementary Fig. 8. Transmission electron microscopy-based assay of the endodermal Casparian strips. The results indicated that the Casparin strip formed at a endodermal cell-cell contact site close to the epicycle face both in wild type **(a)** and *ZmESBL*^{crispr}*-1* mutant **(b,c)**. The results also showed that *ZmESBL*^{crispr}*-1* mutant formed incomplete and less organized Casparin strips, which expanded to a larger region at the later developmental stage (4.0 cm to root apex) **(c)**. The images in **a-c** were representative results of at least three independent replicates with similar results. Source data are provided as a Source Data file.

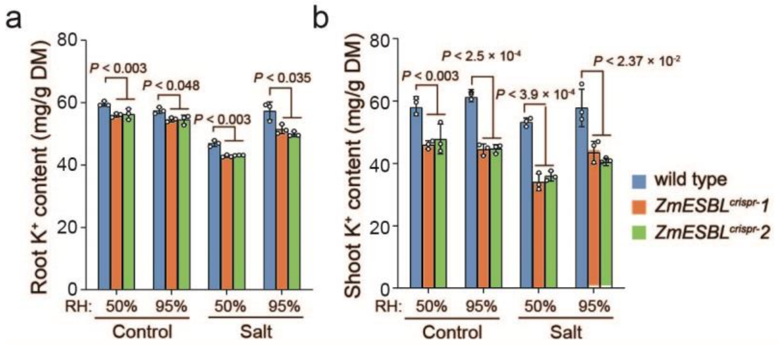

Supplementary Fig. 9. The root (a) and shoot (b) K^+ contents in the wild-type and *ZmESBL*^{crispr} plants. The plants were grown under the indicated conditions, and the results were means ± s.d. of three independent experiments. Statistical significances were determined by a two-sided *t*-test. Source data are provided as a Source Data file.

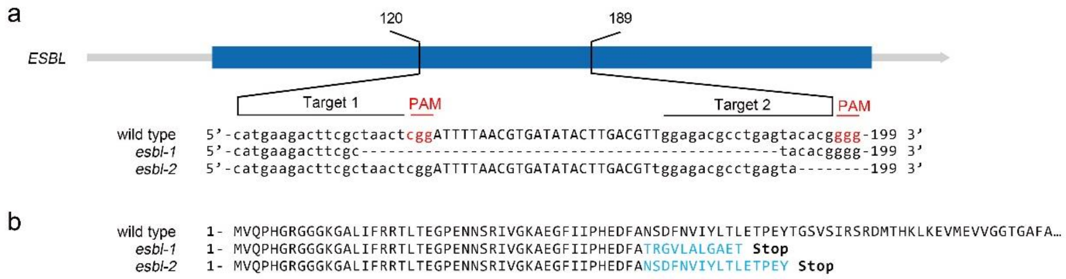

Supplementary Fig. 10. Targeted mutagenesis of Arabidipsis *ESBL* using CRISPR-Cas9-based approach. (a) Mutations in two independent lines (*esbl-1* and *esbl-2*) were shown. PAM sequences were labeled in red. **(b)** Alignment of the amino acid sequences of ESBL coding by indicated *ESBL* alleles. Only the sequences flanking the mutations have been shown. The frameshifted sequences in *esbl-1* and *esbl-2* were highlighted in blue color. Source data are provided as a Source Data file.

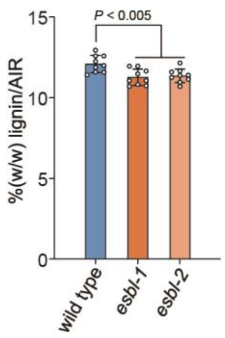

Supplementary Fig. 11. Comparison of lignin content of the *esbl* and wild-type plants. The plants with the indicated genotypes were grown in 1/2 MS medium for 10 days, and then the root tissues were collected to measure the lignin content using the acetyl bromide assay[1]. The results were means ± s.d. of 9 independent experiments. Statistical significances were determined by a two-sided *t*-test. Source data are provided as a Source Data file.

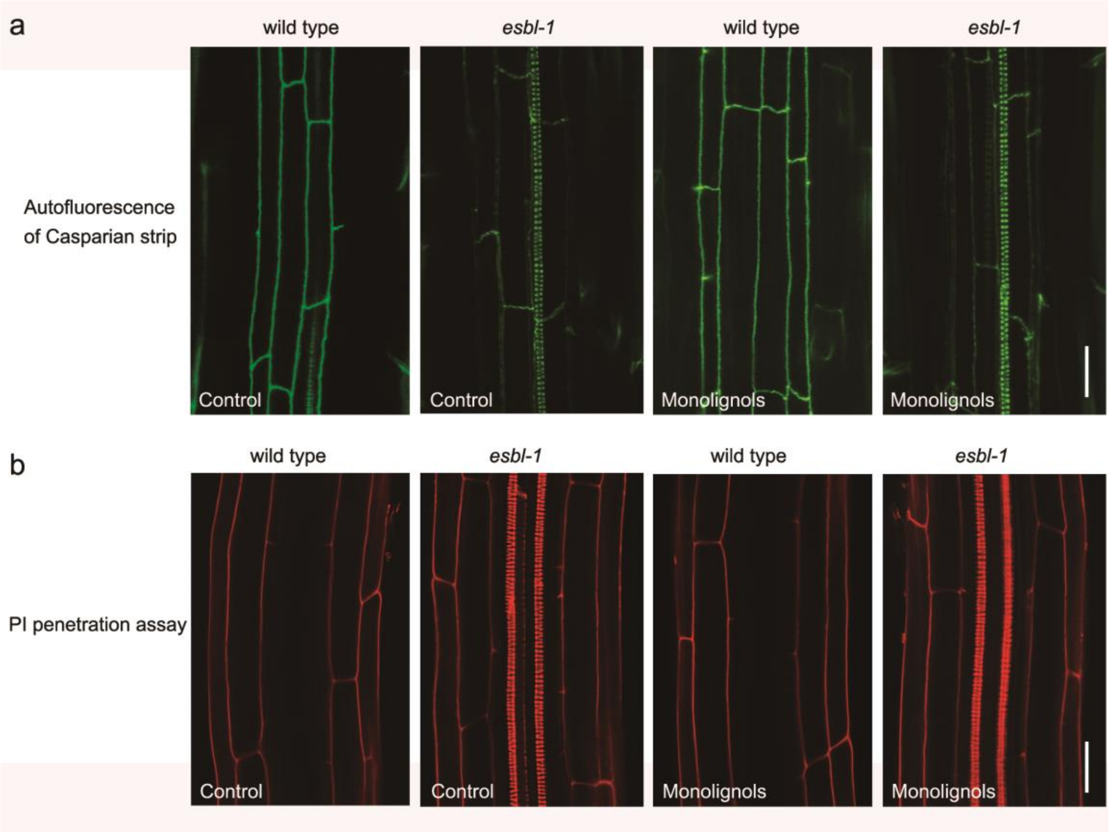

Supplementary Fig. 12. The effect of monolignol treatment on the endodermal CS barrier in the wild-type and *esbl-1* plants. (a) Confocal z-projections of endodermal Casparian strip autofluorescence in the primary root of wild-type and *esbl-1* plants with the indicated treatments. **(b)** PI penetration-based assay of the function of the endodermal CS barrier in indicated samples. The images were taken at about the 25th endodermal cell after the onset of elongation. The results indicated that the monolignols (20 μM coniferyl alcohol plus 20μM sinapyl alcohol) application can neither restore the CS structure **(a)** nor rescue the function of the endodermal barrier **(b)** in *esbl-1* mutant. The images in **a** and **b** were representative of three independent repeats with similar results. Bars = 20 μm. Source data are provided as a Source Data file.

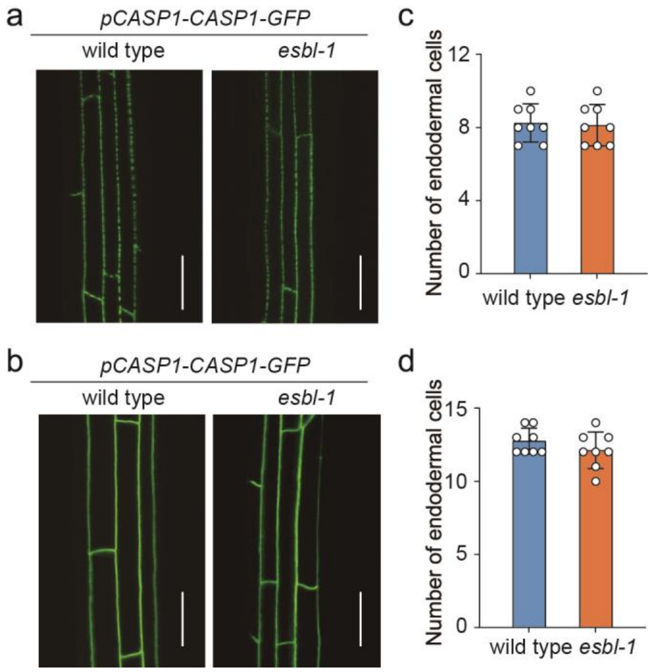

Supplementary Fig. 13. The subcellular localization of CASP1-GFP in the wild-type and *esbl-1* background. The discontinuous **(a)** and continuous **(b)** CASP1-GFP were observed in both wild-type and *esbl-1* plants, and were observed at the same root developmental stage reflected by the number of endodermal cells from the onset of cell elongation **(c, d)**. The images in **a** and **b** were representative of three independent repeats with similar results. Data in **c** and **d** were means ± s.d of 8 roots from three independent batches of seedlings. Bars = 20 μm. Source data are provided as a Source Data file.

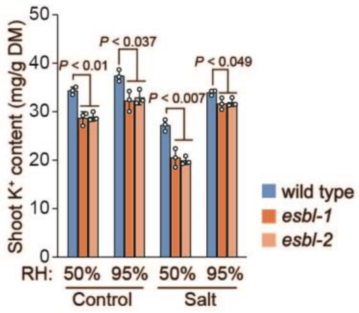

Supplementary Fig. 14. The shoot K^+ contents of *esbl* and wild-type plants with the indicated treatments. The results were means ± s.d. of three independent experiments. Statistical significances were determined by a two-sided *t*-test. Source data are provided as a Source Data file.

Supplementary Table 1. The list of the primers used in this study.

Primers for the fine mapping of *ZmESBL*	
M1-F	GTTGTCACCACTCATCACCG
M1-R	AGCGGCAAGACTTTCTCTCT
M2-F	AGTGTGTGGAGACTGGAGAG
M2-R	AATGAAAACTCGGCCCAAGA
M3-F	TCCATCCCAAAGTCTTCAATCT
M3-R	GGAAGTCAGCATCGAGAGGT
M4-F	GCTCATGATCCATTAGCGCC
M4-R	GACTGTCCGCCAATCATGAC
M5-F	ACGGTACCATCTCGCTTTCA
M5-R	TGGAGGGCTGCATCAACTAT
M6-F	TGACTCAAACACAAGGGCAT
M6-R	TGTCTTCCTCTTGAGGTGG
M7-F	AAGAACTGTGGGTAGCGACA
M7-R	CGAAATGTTTTCCGTGCCAG
M8-F	ACTCATGCCTGTCTCGGAAA
M8-R	TTCCTTTGTTTTCCGTTGGC
M9-F	AGGGTCCCTTTTATAGCCCC
M9-R	AATGCACTTTCAGGTCGACG
M10-F	ATCCTCTGCAACGACGTACT
M10-R	CAATTCCGAACAGTGCCCAT
M11-F	AGCTTGATGTACGAACGCAC
M11-R	CTGCTTTCCTCGCCCTTCATC
Primers for the amplification of *Zm00001d033942/ZmESBL*	
033942-G-F	ACCTGCGATTCCTGAAATGC
033942-G-R	CACGTTGCCAATTCAAATCACA
Primers for the qRT-PCR analysis of *ZmESBL*	
ZmUBI-2-F	TGGTTGTGGCTTCGTTGGTT
ZmUBI-2-R	GCTGCAGAAGAGTTTTGGGTACA
ZmESBL-Q-F	TGTTCCACCACCGGATGACG
ZmESBL-Q-R	GTCAGGTGCACCGTGTCGAA
Primers for the in-situ PCR analysis of *ZmESBL* expression	
Zm18S-in-situ-F	CCATCCCTCCGTAGTTAGCTTCT
Zm18S-in-situ-R	CCTGTCGGCCAAGGCTATATAC
ZmESBL-in-situ-F	TGTTCCACCACCGGATGACG
ZmESBL-in-situ-R	GTCAGGTGCACCGTGTCGAA
Primers for the generation of pAtESBl-GFP-ZmESBL construct	
pESBl-F	ACGCTCTTCTAGTATAAGATCGATGAGAATGAT
pESBl-R	ACGCTCTTCTCATGTGCTGGCTGGACCGGCGAG
GFP-F	ACGCTCTTCTATGGTGAGCAAGGGCGAGGAGCT
GFP-R	ACGCTCTTCTCATCTTGTACAGCTCGTCCATGC
ZmESBL-F	ACGCTCTTCTATGGTCCAACCTCACGGCAGAGG
ZmESBL-R	ACGCTCTTCTCCACTAATGGGCGATCAGCTGAT

Supplementary Table 2. The sequences of *ZmESBL*$^{3H-2}$ and *ZmESBL*^{CIMBL45}.

***ZmESBL*$^{3H-2}$**

TGGATTAAGTAGCGCTGATTCAACTGCTCTCCGAGCATCTTTCTCCGAACCCATCGT
CGTCTGAACAAAATCCAAGAAATCACAACCGATCACCGCTCCGTCGCACCTGCGA
TTCCTGAAATGCTCACCGGGCAGCTGCCCGCTCCCGCATTTGGCCACCACAGATAA
GAAATGATACGACG**ATGCTCGCCAGGATCATCTTCTGCGTCGTCGTCGCCGCC**
GCCGTCCTCGCCGTCGTCCTCCTTGCCACCGTCTCCCCGCTGCCGCACCGGT
CGGGCAGCCGCAAGGGCGCTGCAGGCCAGCGCACCTTCACGGTGTACATCC
ATCCCACGGCATCGACAGCCGTGCGGCAGGAGCAAGCCCAAGGGCGCCGGC
AAGAAGCGAGCGCGCTGGTGTTCCACCACCGGATGACGGCGGGGCCGGAGA
GCGCGTCGAGGGCCGTCGGCGCGGCCTCTGGGTTTCTGCTCCCGGCGGGCG
AGCGGGGTAGTGCCGTGGCGTCGGTGTTCGACACGGTGCACCTGACGTTCG
ACGGCAGCGCCGGGCTGTCCGGCAGTCTCTGCGTCGAGGCGAGCAACGAGA
AGGCGCGCCGGGCCGCGAGACGCCGGCACGGCGGGGAGGAGGAGGAGGTG
CTGCGAGTGGTGGGCGGCACTGGCGCGTTCTCGTTCGCGCGAGGGCATGCC
GTCCTGCGGGGGCAGTGGTCCCGCCCCGGCGCCACGGCGGCGGCGCTTCTT
CTTGAGCTGAGCATTTTCTCTGCTGAGAGTGATCAGCTGATCGCCCATTAGAA
AAGAAAAGAAAAGAATCGGAGCGATCAGTATGTCATAATAAACGATCCA

***ZmESBL*^{CIMBL45}**

TGGATTAAGTAGCGCTGATTCAACTGCTCTCCGAGCATCTTTCTCCGAACCCATCGT
CGTCTGAACAAAATCCAAGAAATCACAACCGATCACCGCTCCGTCGCACCTGCGA
TTCCTGAAATGCTCACCGGGCAGCTGCCCGCTCCCGCATTTGGCCACCACAGATAA
GAAATGATACGACG**ATGCTCGCCAGGATCATCTTCTGCGTCGTCGTCGCCGCC**
GCCGTCCTCGCCGTCGTCCTCCTTGCCACCGTCTCCCCGCTGCCGCACCGGT
CGGGCAGCCGCAAGGGCGCTGCAGGCCAGCGCACCTTCACGGTGTACATCC
ATCCCACGGCATCGACAGCCGTGCGGCAGGAGCAAGCCCAAGGGCGCCGGC
AAGAAGCGAGCGCGCTGGTGTTCCACCACCGGATGACGGCGGGGCCGGAGA
GCGCGTCGAGGGCCGTCGGCGCGGCCTCTGGGTTTCTGCTCCCGGCGGGCG
AGCGGGGTAGTGCCGTGGCGTCGGTGTTCGACACGGTGCACCTGACGTTCG
ACGGCAGCGCCGGGCTGTCCGGCAGTCTCTGCGTCGAGGCGAGCAACGAGA
AGGCGCGCCGGGCCGCGAGACGCCGGCACGGCGGGGAGGAGGAGGAGGTG
CTGCGAGTGGTGGGCGGCACTGGCGCGTTCTCGTTCGCGCGAGGGCATGCC
GTCCCGCGAGGGCA(Insertion-T)GCCGTCCCGCCCCGGCGCCACGGCGGCGG

SNP503 SNP507 Ins512 SNP514 SNP515

CGCTTCTTCTTGAGCTGAGCATTTTCTCTGCTGAGAGTGATCAGCTGATCGC
CCATTAGAAAAGAAAAGAAAAGAATCGGAGCGATCAGTATGTCATAATAAACGAT
CCA

Supplementary references

1. Xiao, C., Barnes, W.J., Zamil, M.S., Yi, H., Puri, V.M. & Anderson, C.T. Activation tagging of Arabidopsis POLYGALACTURONASE INVOLVED IN EXPANSION2 promotes hypocotyl elongation, leaf expansion, stem lignification, mechanical stiffening, and lodging. *Plant J*. **89,** 1159-1173 (2017).
